# The role of visual rating and automated brain volumetry in early detection and differential diagnosis of Alzheimer's disease

**DOI:** 10.1111/cns.14492

**Published:** 2023-10-21

**Authors:** Yingren Mai, Zhiyu Cao, Lei Zhao, Qun Yu, Jiaxin Xu, Wenyan Liu, Bowen Liu, Jingyi Tang, Yishan Luo, Wang Liao, Wenli Fang, Yuting Ruan, Ming Lei, Vincent C. T. Mok, Lin Shi, Jun Liu, for the Alzheimer's Disease Neuroimaging Initiative

**Affiliations:** ^1^ Department of Neurology The Second Affiliated Hospital of Guangzhou Medical University Guangzhou China; ^2^ Department of Neurology, Sun Yat‐sen Memorial Hospital Sun Yat‐sen University Guangzhou China; ^3^ BrainNow Research Institute Shenzhen China; ^4^ Department of Statistics, College of Liberal Art and Sciences University of Illinois Urbana‐Champaign Urbana Illinois USA; ^5^ Department of Rehabilitation The Second Affiliated Hospital of Guangzhou Medical University Guangzhou China; ^6^ Division of Neurology, Department of Medicine and Therapeutics, Gerald Choa Neuroscience Centre, Lui Che Woo Institute of Innovative Medicine The Chinese University of Hong Kong Hong Kong, SAR China; ^7^ Department of Imaging and Interventional Radiology The Chinese University of Hong Kong Hong Kong, SAR China

**Keywords:** Alzheimer's disease, automated brain volumetry, medial temporal lobe atrophy scale, mild cognitive impairment, quantitative MTA, structural magnetic resonance imaging

## Abstract

**Background:**

Medial temporal lobe atrophy (MTA) is a diagnostic marker for mild cognitive impairment (MCI) and Alzheimer's disease (AD), but the accuracy of quantitative MTA (QMTA) in diagnosing early AD is unclear. This study aimed to investigate the accuracy of QMTA and its related components (inferior lateral ventricle [ILV] and hippocampus) with MTA in the early diagnosis of MCI and AD.

**Method**s**:**

This study included four groups: normal (NC), MCI stable (MCIs), MCI converted to AD (MCIs), and mild AD (M‐AD) groups. Magnetic resonance image analysis software was used to quantify the hippocampus, ILV, and QMTA. MTA was rated by two experienced neurologists. Receiver operating characteristic area under the curve (AUC) analysis was performed to compare their capability in differentiating AD from NC and MCI, and optimal thresholds were determined using the Youden index.

**Results:**

QMTA distinguished M‐AD from NC and MCI with higher diagnostic accuracy than MTA, hippocampus, and ILV (AUC_NC_ = 0.976, AUC_MCI_ = 0.836, AUC_MCIs_ = 0.894, AUC_MCIc_ = 0.730). The diagnostic accuracy of QMTA was superior to that of MTA, the hippocampus, and ILV in differentiating MCI from AD. The diagnostic accuracy of QMTA was found to remain the best across age, sex, and pathological subgroups analyzed. The sensitivity (92.45%) and specificity (90.64%) were higher in this study when a cutoff value of 0.635 was chosen for QMTA.

**Conclusions:**

QMTA may be a better choice than the MTA scale or the associated quantitative components alone in identifying AD patients and MCI individuals with higher progression risk.

## Graphical abstract

The quantitative visual score medial temporal lobe atrophy index (QMTA) can be utilized as a clinical diagnostic tool for Alzheimer's disease. When the QMTA value is 0.635 or higher, it may indicate probable Alzheimer's disease.

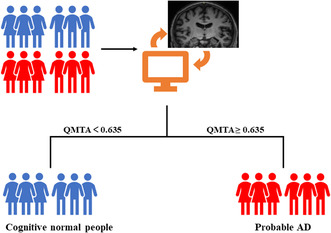

## 1 INTRODUCTION

Alzheimer's disease (AD) is a multifactorial neurodegenerative disease.^1^ AD patients initially exhibit mild cognitive impairment, early AD includes mild cognitive impairment (MCI) to mild dementia, early AD includes mild cognitive impairment (MCI) to mild dementia.^2^ With the accumulation of AD pathology, brain neurons are progressively lost. It is difficult to regenerate nerves in the elderly, early prevention of pathological development may achieve better results, but the heterogeneity of MCI and AD leads to significant diagnostic challenges.^3^ The annual conversion rate of MCI to AD progression is 10%–15%, with a cumulative conversion rate of more than 50% over 5 years.^4^, ^5^ Although confirmatory diagnostic tools such as cerebrospinal fluid (CSF) and positron emission tomography can detect AD‐associated β‐amyloid (Aβ) and tau pathology, they have disadvantages such as invasiveness, radiation exposure, high cost, poor accessibility,^6^ and low predictive accuracy and specificity.^7^, ^8^ The early stages are the best time for AD treatment, but the heterogeneity of MCI and AD leads to significant diagnostic challenges.^9^

Brain atrophy is a neurodegenerative change closely associated with cognitive changes in AD.^10^ Structural magnetic resonance imaging (sMRI) is a non‐invasive tool for routine clinical diagnosis because it can visualize the severity of brain atrophy,^11^, ^12^ but subtle and overall changes in brain structure are difficult to identify by MRI. With the development of machine learning, image quantification results calculated using sMRI can be derived into comprehensive and clinically feasible diagnostic metrics, such as the AD‐RAI in AccuBrain®.^13^, ^14^

Hippocampal volumes (HV) are one of the most important biomarkers of AD, but its measurement is cumbersome, and assessment is easily influenced by cranial size.^15^ The Medial Temporal Atrophy (MTA) scale is a recognized semi‐quantitative tool for visually assessing the grade of hippocampal atrophy,^16^, ^17^ which distinguishes AD from normal aging and has 77% accuracy in predicting the conversion of MCI to AD.^18^ Automated computational tools for quantifying brain structures allow for efficient and specific acquisition of structural brain volumes.^19^ The hippocampal occupancy score is an index used to estimate hippocampal atrophy based on HV occupancy and to predict the risk of early AD progression.^20^ Quantitative MTA (QMTA) is based on hippocampal and inferior lateral ventricle (ILV) volumes, and can assess relative hippocampal atrophy to mimic the visual rating logic of the MTA scale. Preliminary studies have found QMTA to be 90% accurate in differentiating AD from controls and provide a better objective and quantitative assessment of the degree of medial temporal lobe atrophy.^14^ However, no studies have yet elucidated its performance compared with MTA in identifying early AD and predicting the risk of MCI progression.

The main objective of this study was to compare the diagnostic efficiency of QMTA and its related components (ILV and hippocampal volume) with MTA visual scores for the early diagnosis of MCI and AD. The secondary goal was to further evaluate the accuracy of QMTA and MTA in predicting conversion to AD in patients with MCI, which will determine the accuracy of the QMTA optimal diagnostic threshold in differentiating early AD.

## 2 METHODS

### 2.1 Study participants

All data were acquired from the Alzheimer's Disease Neuroimaging Initiative (ADNI) database (http://adni.loni.usc.edu).^21^ The ADNI database was established in 2003 and has been extensively reviewed elsewhere (http://www.adni‐info.org).

As shown in Table 1 1696 subjects were included in this study, of which 534 were in the NC group, 884 were in the MCI group (including 573 MCI stable and 311 MCI converters), and 278 were in the AD group. All participants underwent neuropsychological cognitive assessment and 3D T1W MRI scanning at baseline. The NC group had no clinical symptoms of cognitive impairment, with a Mini‐Mental State Scale (MMSE) score ≥ 26 and Clinical Dementia Rating (CDR) = 0. MCI patients had normal activities of daily living, with an MMSE score ≥ 24 and CDR < 0.5. The diagnostic information during the follow‐up period of the enrolled study subjects was also considered, and MCI patients were subdivided into two groups: MCI stable (MCIs, remaining MCI during the follow‐up period) and MCI converters (MCIc, converted to AD during the follow‐up period). AD patients met the diagnostic criteria of the National Institute of Neurological and Communicative Disorders and Stroke and the Alzheimer's Disease and Related Disorders Association (NINCDS/ADRDA) for probable AD. We further used neuropsychological scale assessment criteria to classify the degree of AD dementia, in which those with MMSE scores of 20–26 and CDR of 0.5–1 in the AD group were defined as M‐AD.

**TABLE 1:** Demographic characteristics, brain structure, and cognitive performance across NC, MCI, and AD groups.

	*N*	NC (*n* = 534)	MCIs (*n* = 573)	MCIc (*n* = 311)	M‐AD (*n* = 278)
Age (years), median (IQR)	1696	72.25 (68.00, 77.00)	73.00 (67.00, 78.00)	74.00 (69.00, 79.00)*,**	75.20 (70.00, 80.00)*,**
Male, *n* (%)	1696	228 (42.70)	319 (55.67)*	190 (61.09)*,**	163 (58.63)*,**,***
Education (years), median (IQR)	1696	16.00 (15.00, 18.00)	16.00 (14.00, 18.00)*	16.00 (14.00, 18.00)*	16.00 (12.00, 17.00)*,**,***
MMSE, median (IQR)	1696	29.00 (29.00, 30.00)	28.00 (27.00, 29.00)	27.00 (26.00, 29.00)*,**	23.00 (21.00, 25.00)*,**,***
FAQ, median (IQR)	1678	0.00 (0.00, 0.00)	1.00 (0.00, 3.00)*	4.00 (1.00,8.00)*,**	13.00 (9.00, 18.00)*,**,***
CDR, median (IQR)	1696	0.00 (0.00, 0.00)	0.50 (0.50, 0.50)*	0.50 (0.50, 0.50)*	1.00 (0.50, 1.00)*,**,***
CDR‐SOB, median (IQR)	1696	0.00 (0.00, 0.00)	1.00 (0.50, 2.00)*	1.50 (1.00, 2.50)*,**	4.50 (3.50, 5.13)*,**,***
ADAS‐cog, median (IQR)	1696	7.33 (5.00, 10.00)	10.00 (7.00, 14.00)*	17.33 (12.00, 22.00)*,**	25.33 (20.85, 30.67)*,**,***
CSF Aβ_42_ (pg/mL), median (IQR)	1131	1529.00 (1182.00, 2022.00)	1077.00 (736.50, 1633.50)*	669.90 (553.00, 863.25)*,**	600.00 (462.00, 749.00)*,**
CSF P‐tau^181^ (pg/mL), median (IQR)	1122	19.00 (15.43, 24.79)	20.80 (15.93, 28.52)*	32.00 (24.43, 46.42)*,**	32.70 (26.36, 40.96)*,**
CSF T‐tau (pg/mL), median (IQR)	1122	215.45 (176.90, 270.95)	233.60 (180.50, 302.75)	326.00 (252.00, 434.10)*	325.60 (271.10, 400.55)*,**
HV (mL), mean ± SD	1696	6.36 ± 0.74	6.03 ± 0.88*	5.44 ± 0.93*,**	4.97 ± 0.93*,**,***
HF, mean ± SD	1696	0.432 ± 0.046	0.403 ± 0.059*	0.359 ± 0.057*,**	0.330 ± 0.057*,**,***
ILV volume(mL), median (IQR)	1696	2.81 (2.35, 3.34)	3.24 (2.58, 4.03)*	4.08 (3.16, 5.24)*,**	5.17 (4.07, 6.49)*,**,***
ILV fraction, median (IQR)	1696	0.188 (0.165, 0.222)	0.214 (0.178, 0.259)*	0.267 (0.212, 0.333)*,**	0.342 (0.277, 0.418)*,**,***
QMTA, median (IQR)	1696	0.435 (0.373, 0.528)	0.528 (0.410, 0.701)*	0.749 (0.565, 1.010)*,**	1.080 (0.797, 1.340)*,**,***
MTA‐avg, median (IQR)	1696	1.00 (0.50, 1.00)	1.00 (1.00, 2.00)*	2.00 (1.00, 2.50)*,**	2.50 (2.00, 3.00)*,**,***

*Note*: With significant difference from *NC (*p* < 0.05); **MCIs (*p* < 0.05)；***MCIc (*p* < 0.05).

Abbreviations: ADAS‐cog, Alzheimer's disease assessment scale‐cognitive; avg, average; CDR, clinical dementia rating; CDR‐SOB, clinical dementia rating sum of boxes; FAQ, Functional Activities Questionnaire; HF, hippocampal fraction; HV, the absolute volumes of hippocampus; ILV, inferior lateral ventricle; IQR, interquartile range.

### 2.2 CSF biomarkers

Some of the participants underwent baseline CSF tests. The collection and processing methods are described at http://www.adni‐info.org/. The Aβ42 classification criteria were as follows: if Aβ1‐42 ≤ 880 pg/mL, CSF Aβ1‐42 was considered positive.^22^ According to the 2018 NIA‐AA research framework, A/T/N AD‐related biological diagnostic criteria include amyloid (A), pathological tau (T), and neurodegeneration (N).^23^ The MCI group was divided into two subgroups: MCI with CSF Aβ_42_ negative (MCI A‐) and MCI with CSF Aβ_42_ positive (MCI A+).

### 2.3 MR imaging

The 3D T1W MRI data of 1696 subjects scanned by Philips, Siemens, and GE MR scanners at baseline were included. MRI data were acquired using a standardized ADNI MRI protocol. We downloaded the preprocessed T1W MRI images after prescaling, intensity nonuniform correction, and distortion correction from the ADNI database for subsequent quantitative and visual rating analysis.

### 2.4 Visual assessment

Visual rating of MTA was performed on the coronal T1W image parallel to the brainstem axis and through the hippocampus at the level of the anterior pons. Both hemispheres were assessed using the 0–4 scoring system of MTA.^24^ The average MTA value on the left and right sides is the final MTA score (MTA‐avg) of the subject.

We used the intraclass correlation coefficient (ICC) to measure the inter‐rater reliability of visual scores. MR images of 100 subjects were randomly selected for visual rating of MTA and the assessors were blinded to clinical and demographic information about the subjects. Four neurologists (MYR, CZY, YQ, and XJX) conducted the MTA rating and among them, MYR and CZY were with rich experience in visual scoring. As described previously,^25^ two‐way random, absolute, single‐measure ICCs [ICC (1, 2)] were used to evaluate the reliability of each scale at the level of each rater. Average measures ICCs [ICC (2, k)] were used to evaluate the improvement in reliability of the scale based on average scores from all raters. Both for single‐measures ICCs and average measures ICCs, the highest consistency was found for the combined assessment of MYR and CZY [ICC (1, 2) = 0.908 (0.867, 0.937); ICC (2, k) = 0.952 (0.929–0.968)]. When performed by four raters, the ICCs were reduced [ICC (1, 2) = 0.821 (0.768, 0.867); ICC (2, k) = 0.948 (0.930, 0.963)]. Therefore, we used the mean scores of the two assessors (MYR and CZY) as the patient's final MTA score. When visually assessing all 1696 subjects, MYR and CZY still reached a high ICC of almost 0.9 (see Table S1 for details of the results).

### 2.5 MR image processing

The 3D T1W MRI of the subjects was processed with AccuBrain®, a brain quantification tool (cloud‐based commercial system with FDA approval and CE mark) that performs fully automatic quantification of brain structure and tissue. The absolute HV and ILV were calculated from the corresponding segmentations, and they were normalized by the intracranial volume (ICV) to generate the relative volumes, that is, the hippocampal fraction (HF, % of ICV) and ILV fraction (% of ICV). The QMTA is a quantitative indicator used to reflect MTA. Similar to the logic of MTA assessment (enlargement of the ILV and atrophy of the hippocampus), QMTA was calculated from the volume ratio of the ILV to the hippocampus.

$$\text{QMTA}=\frac{\mathrm{ILV}\;\text{volume}\;\left(L\right)+\mathrm{ILV}\;\text{volume}\;\left(R\right)}{\text{hippocampus volume}\;\left(L\right)+\text{hippocampus volume}\;\left(R\right)}$$

### 2.6 Statistical analysis

For variables that exhibit normal distribution, we use mean ± SD to describe and use parametric test methods. Otherwise, we use the median and interquartile range (IQR) to describe, and use nonparametric test methods. One‐way analysis of variance with post‐hoc Bonferroni correction for multiple comparisons was performed to evaluate the differences in subject characteristics, brain volumetric measures, and CSF‐based measures. The chi‐squared test was used to determine differences in categorical variables between the groups. Correlation analysis was performed using Spearman's correlation coefficient. The ICC two‐way random‐effects model was used to evaluate the reliability and consistency of the MTA visual rating results between the two visual raters.^26^

We used the receiver operating characteristic (ROC) area under the curve (AUC) with DeLong's test to assess the diagnostic performance of the brain structural indices. Sub‐analyses were conducted by grouping the study cohort by age (>75 years or not), sex, and status of ATN‐based pathological diagnosis.

Statistical significance was set at *p* < 0.05 (two‐sided). Statistical analyses were performed using SPSS Statistics version 25.0.0, MedCalc, and R version 4.1.2.

## 3 RESULTS

### 3.1 Demographic characteristics

As shown in Table 1, compared with the other three groups, the QMTA, MTA, hippocampus, and ILV of the AD group were statistically different (*p* < 0.001). There were also significant differences in brain structure indicators in the MCIc group compared with the MCIs or NC groups (*p* < 0.001). Demographic information of the A/T/N subgroups is presented in Table S2.

### 3.2 Effects of clinical characteristics, CSF biomarkers, and brain structure on QMTA and MTA

A significant correlation was observed between QMTA and MTA (*R* = 0.832; *p* < 0.001). We analyzed the correlation between QMTA, MTA, cognition, CSF biomarkers, and brain structure (Table 2). We found that QMTA was significantly correlated with cognition (*R*_MMSE_ = −0.533, *R*_ADAS‐cog_ = 0.615; all, *p* < 0.001), and the value of the correlation was numerically higher than that of MTA (*R*_MMSE_ = −0.469, *R*_ADAS‐cog_ = 0.527; all *p* < 0.001). QMTA also correlated with cognitive severity (*R*_CDR_ = 0.529, *R*_CDR‐SOB_ = 0.572; all *p* < 0.001). In addition, QMTA and MTA were strongly correlated with Aβ_42_ (*R*_QMTA_ = −0.533, *R*_MTA‐avg_ = −0.448; all *p* < 0.001) and weakly correlated with P‐tau^181^ (*R*_QMTA_ = 0.244, *R*_MTA‐avg_ = 0.222; *p* < 0.001) and T‐tau (*R*_QMTA_ = 0.211, *R*_MTA‐avg_ = 0.191; all *p* < 0.001). QMTA and MTA were also significantly correlated with the volume and fraction of the hippocampus and the ILV.

**TABLE 2:** Correlation between MMSE, CSF biomarkers, brain volumetry, MTA, and QMTA.

	Rs (MTA‐avg)	*p*_MTA_	Rs (QMTA)	*p*_QMTA_
MMSE	−0.469	<0.001	−0.533	<0.001
CDR	0.486	<0.001	0.529	<0.001
CDR‐SOB	0.526	<0.001	0.572	<0.001
ADAS‐cog	0.527	<0.001	0.615	<0.001
CSF Aβ_42_	−0.448	<0.001	−0.533	<0.001
CSF P‐tau^181^	0.222	<0.001	0.244	<0.001
CSF T‐tau	0.191	<0.001	0.211	<0.001
Hip volume	−0.507	<0.001	−0.579	<0.001
Hip fraction	−0.593	<0.001	−0.739	<0.001
ILV volume	0.768	<0.001	0.926	<0.001
ILV fraction	0.801	<0.001	0.941	<0.001
QMTA	0.832	<0.001	–	–
MTA‐avg	–	–	0.832	<0.001

Abbreviations: ADAS‐cog, Alzheimer's disease assessment scale‐cognitive; avg, average; CDR, clinical dementia rating; CDR‐SOB, clinical dementia rating sum of boxes; FAQ, Functional Activities Questionnaire; HF, hippocampal fraction; HV, the absolute volumes of hippocampus; ILV, inferior lateral ventricle.

### 3.3 Diagnostic accuracy of all subjects

The diagnostic accuracies of QMTA, MTA, and a single brain structure in distinguishing M‐AD, MCIc, MCIs, and NC are shown in Table 3. The results of the comparison of the AUC values of the different indicators can be found in Table S3.

**TABLE 3:** ROC curve analyses for differentiating different diagnoses with SMRI indexes.

	AUC (95% CI)	Youden index (%)	Optimal threshold	Sensitivity (%)	Specificity (%)
QMTA					
M‐AD vs. NC (*n* = 812)	0.976 (0.963, 0.985)	83.08	≥0.635	92.45	90.64
M‐AD vs. MCI (*n* = 1162)	0.836 (0.814, 0.857)	51.77	≥0.719	85.25	66.52
M‐AD vs. MCIs (*n* = 851)	0.894 (0.871, 0.914)	62.40	≥0.712	85.61	76.79
M‐AD vs. MCIc (*n* = 589)	0.730 (0.692, 0.766)	34.44	≥0.824	73.02	61.41
MCI vs. NC (*n* = 1418)	0.734 (0.710, 0.757)	38.58	≥0.555	56.56	82.02
MCIs vs. NC (*n* = 1107)	0.662 (0.633, 0.689)	27.57	≥0.555	45.55	82.02
MCIc vs. NC (*n* = 845)	0.866 (0.842, 0.889)	59.27	≥0.552	77.81	81.46
MCIc vs. MCIs (*n* = 884)	0.725 (0.694, 0.754)	33.87	≥0.635	67.20	66.67
MTA scale (average)					
M‐AD vs. NC (*n* = 812)	0.937 (0.918, 0.952)	75.09	≥1.50	86.33	88.76
M‐AD vs. MCI (*n* = 1162)	0.799 (0.775, 0.822)	47.30	≥1.50	86.33	60.97
M‐AD vs. MCIs (*n* = 851)	0.853 (0.827, 0.876)	56.49	≥1.50	86.33	70.16
M‐AD vs. MCIc (*n* = 589)	0.701 (0.662, 0.738)	30.76	≥2.00	62.59	68.17
MCI vs. NC (*n* = 1418)	0.703 (0.679, 0.727)	31.18	≥1.00	53.28	77.90
MCIs vs. NC (*n* = 1107)	0.646 (0.617, 0.675)	21.18	≥1.00	43.28	77.90
MCIc vs. NC (*n* = 845)	0.808 (0.780, 0.834)	49.61	≥1.00	71.70	77.90
MCIc vs. MCIs (*n* = 884)	0.682 (0.650, 0.713)	28.42	≥1.00	71.70	56.72
Hippocampal volume (mL)					
M‐AD vs. NC (*n* = 812)	0.881 (0.857, 0.903)	60.84	≤5.81	82.37	78.46
M‐AD vs. MCI (*n* = 1162)	0.743 (0.717, 0.768)	37.54	≤5.14	59.71	77.83
M‐AD vs. MCIs (*n* = 851)	0.801 (0.772, 0.827)	47.43	≤5.56	75.18	72.25
M‐AD vs. MCIc (*n* = 589)	0.637 (0.597, 0.676)	23.06	≤5.14	59.71	63.34
MCI vs. NC (*n* = 1418)	0.668 (0.643, 0.693)	27.45	≤5.81	48.98	78.46
MCIs vs. NC (*n* = 1107)	0.605 (0.575, 0.634)	16.93	≤5.93	42.58	74.34
MCIc vs. NC (*n* = 845)	0.786 (0.757, 0.813)	47.57	≤5.77	68.17	79.40
MCIc vs. MCIs (*n* = 884)	0.691 (0.659, 0.721)	33.76	≤5.59	62.38	71.38
Hippocampal fraction (%)					
M‐AD vs. NC (*n* = 812)	0.920 (0.899, 0.937)	68.02	≤0.369	76.26	91.76
M‐AD vs. MCI (*n* = 1162)	0.755 (0.729, 0.780)	38.70	≤0.369	76.26	62.44
M‐AD vs. MCIs (*n* = 851)	0.817 (0.789, 0.842)	50.26	≤0.369	76.26	74.00
M‐AD vs. MCIc (*n* = 589)	0.642 (0.601, 0.680)	22.42	≤0.329	50.72	71.70
MCI vs. NC (*n* = 1418)	0.714 (0.690, 0.737)	33.56	≤0.414	66.52	67.04
MCIs vs. NC (*n* = 1107)	0.645 (0.616, 0.673)	23.93	≤0.414	56.89	67.04
MCIc vs. NC (*n* = 845)	0.840 (0.814, 0.864)	54.16	≤0.396	75.88	78.28
MCIc vs. MCIs (*n* = 884)	0.709 (0.678, 0.739)	32.84	≤0.369	58.84	74.00
ILV volume (mL)					
M‐AD vs. NC (*n* = 812)	0.928 (0.908, 0.945)	69.53	≥3.47	89.57	79.96
M‐AD vs. MCI (*n* = 1162)	0.787 (0.763, 0.810)	43.65	≥4.34	69.78	73.87
M‐AD vs. MCIs (*n* = 851)	0.840 (0.814, 0.864)	52.73	≥4.23	71.58	81.15
M‐AD vs. MCIc (*n* = 589)	0.690 (0.651, 0.727)	27.98	≥4.34	69.78	58.20
MCI vs. NC (*n* = 1418)	0.691 (0.666, 0.715)	30.66	≥3.93	52.94	77.72
MCIs vs. NC (*n* = 1107)	0.635 (0.606, 0.663)	23.61	≥3.39	45.90	77.72
MCIc vs. NC (*n* = 845)	0.794 (0.765, 0.821)	45.25	≥3.82	57.23	88.01
MCIc vs. MCIs (*n* = 884)	0.675 (0.643, 0.706)	27.62	≥3.86	56.59	71.03
ILV fraction (%)					
M‐AD vs. NC (*n* = 812)	0.947 (0.929, 0.961)	72.63	≥0.232	91.73	80.90
M‐AD vs. MCI (*n* = 1162)	0.815 (0.791, 0.837)	48.53	≥0.263	82.01	66.52
M‐AD vs. MCIs (*n* = 851)	0.868 (0.843, 0.890)	58.45	≥0.263	82.01	76.44
M‐AD vs. MCIc (*n* = 589)	0.717 (0.679, 0.753)	31.70	≥0.280	74.46	57.23
MCI vs. NC (*n* = 1418)	0.693 (0.668, 0.717)	30.44	≥0.211	60.41	70.04
MCIs vs. NC (*n* = 1107)	0.633 (0.604, 0.662)	22.92	≥0.203	57.94	64.98
MCIc vs. NC (*n* = 845)	0.803 (0.774, 0.829)	47.70	≥0.241	63.99	83.71
MCIc vs. MCIs (*n* = 884)	0.686 (0.654, 0.716)	30.70	≥0.246	61.41	69.28

Abbreviations: AUC, area under the curve; CI, confidence interval; ILV, inferior lateral ventricle; MTA scale, visual rating of medial temporal lobe atrophy (average score of left and right hemispheres); vs., versus.

In distinguishing M‐AD from NC, QMTA showed higher accuracy than MTA, HV/HF, and ILV (AUC_QMTA_ = 0.976, AUC_MTA‐avg_ = 0.937, AUC_HV_ = 0.881, AUC_HF_ = 0.920, AUC_ILVvolume_ = 0.928, AUC_ILVfraction_ = 0.947, all *p* < 0.001). Among them, QMTA achieved the best specificity and sensitivity (92.45% and 90.64%, respectively) in distinguishing AD from NC when the cutoff value was 0.635. When distinguishing M‐AD from MCI and MCIs, QMTA also had higher diagnostic accuracy than the quantitative indices of MTA, hippocampus, and ILV (all *p* < 0.001).

When distinguishing NC from MCIs or MCIc, the diagnostic accuracy of QMTA was higher than that of MTA‐avg, the hippocampus, and ILV. The superiority of QMTA was more pronounced in identifying MCIc from MCIs, with an AUC of 0.725, which was significantly higher than that of MTA (AUC = 0.682, *p* < 0.001), ILV volume (AUC = 0.675, *p* < 0.001), and ILV fraction (AUC = 0.686, *p* < 0.001) but not significantly higher than that of HV and HF (AUC_HV_ = 0.691, AUC_HF_ = 0.709; both *p* > 0.05).

### 3.4 Impact of covariates

Although there were significant differences in age, sex, and education between these four groups, the absolute values of the differences were small; thus, we did not adjust the covariates in the primary analyses. To exclude the effects of covariates, we also conducted ROC analysis adjusted for age, sex, and education and found that the pattern of results remained unchanged^27^ (see Tables S4–S6).

### 3.5 Diagnostic accuracy of subgroup analysis

To further analyze the diagnostic accuracy of QMTA, we divided the study subjects into different subgroups for analysis by age, sex, or A/T/N pathology type.^27^, ^28^

#### 3.5.1 Age

We analyzed the accuracy of brain structure diagnosis in age subgroups (see Tables S7 and S8). The results of ≤75 years and >75 years subgroups were similar to the results of the overall population analysis, and the diagnostic accuracy of QMTA was higher than that of MTA, hippocampus, and ILV. To distinguish M‐AD from NC, QMTA had the best diagnostic accuracy (≤75 years AUC = 0.979, >75 years AUC = 0.973). In distinguishing NC from MCIs or MCIc, the diagnostic performance of brain structure indicators in the >75 years group was improved (AUC = 0.700–0.900). In distinguishing MCIs and MCIc, the diagnostic accuracy of QMTA in the >75 years group (AUC = 0.707) was slightly lower than that in other groups.

#### 3.5.2 Gender

Gender subgroups compared structural brain indicators such as hippocampus, lateral subventricular horn, and QMTA (see Table S9). We found that structural brain indexes of ILV, IF, QMTA, and MTA were significantly greater in males than in females, but the hippocampus was significantly atrophied in females. Their diagnostic accuracy (see Tables S10 and S11). Differentiating NC from M‐AD, QMTA (AUC females = 0.978, AUC males = 0.975) was more accurate than other brain structure indexes (all *p* < 0.001). In distinguishing NC from MCIs and MCIc, the accuracy of the distinction was similar to that of the overall analysis. Gender had little effect on the accuracy, specificity, and sensitivity of QMTA's diagnostic performance, but the cutoff values were remarkably different. The cutoff value difference is likely due to the difference hippocampus volume between men and women.^29^, ^30^

#### 3.5.3 A/T/N pathology type

##### NC A− and M‐AD A+

When distinguishing NC A− from M‐AD A+, the diagnostic accuracy of QMTA, MTA, and ILV improved (Table 4), and the diagnostic accuracy of QMTA (AUC = 0.981) remained significantly higher than that of MTA (AUC = 0.939, *p* < 0.001), HV (AUC = 0.864, *p* < 0.001), HF (AUC = 0.918, *p* < 0.001), ILV volume (AUC = 0.944, *p* < 0.001), and ILV fraction (AUC = 0.958, *p* < 0.001). QMTA had a better diagnostic performance than HV (*p* < 0.001), but there was no other single brain structure to better distinguish the amyloid‐positive AD population.

**TABLE 4:** Diagnostic accuracy of different indexes between amyloid‐related subgroups.

	QMTA	MTA‐avg	Hippocampal volume (mL)	Hippocampal fraction (%)	ILV volume (mL)	ILV fraction (%)	*p*
NC A− vs. M‐AD A+							
AUC (95% CI)	0.981 (0.964, 0.991)	0.939 (0.915, 0.959)	0.864 (0.831, 0.893)	0.918 (0.890, 0.941)	0.944 (0.920, 0.963)	0.958 (0.936, 0.974)	a, b, c, d, e, f, j, k, l, n, o
Threshold	≥0.552	≥1.50	≤5.82	≤0.369	≥3.70	≥0.234	/
Sensitivity (%)	97.60	85.63	80.84	74.25	85.03	89.22	/
Specificity (%)	88.00	90.46	79.38	92.62	91.38	87.38	/
NC A− vs. MCIc A+							
AUC (95% CI)	0.883 (0.851, 0.910)	0.813 (0.775, 0.846)	0.796 (0.757, 0.831)	0.849 (0.814, 0.880)	0.795 (0.756, 0.829)	0.808 (0.770, 0.842)	a, b, d, e, j, m
Threshold	≥0.552	≥1.00	≤5.83	≤0.396	≥3.50	≥0.241	/
Sensitivity (%)	77.58	71.52	72.12	78.18	63.03	61.82	/
Specificity (%)	88.00	79.69	78.77	79.08	86.77	88.62	/
MCIs A− vs. MCIc A+							
AUC (95% CI)	0.766 (0.724, 0.805)	0.707 (0.661, 0.749)	0.735 (0.691, 0.776)	0.747 (0.703, 0.787)	0.696 (0.651, 0.739)	0.715 (0.670, 0.756)	a, d, e, o
Threshold	≥0.558	≥1.00	≤5.76	≤0.396	≥3.86	≥0.259	/
Sensitivity (%)	75.76	71.52	69.70	78.18	54.55	55.15	/
Specificity (%)	66.79	63.14	72.63	63.50	76.28	81.39	/

*Note*: a: AUC (QMTA) vs. AUC (MTA‐avg), *p* < 0.05; b: AUC(QMTA) vs. AUC(HV), *p* < 0.05; c: AUC (QMTA) vs. AUC (HF), *p* < 0.05; d: AUC (QMTA) vs. AUC (ILV volume), *p* < 0.05; e: AUC (QMTA) vs. AUC (ILV fraction), *p* < 0.05; f: AUC (MTA‐avg) vs. AUC (HV), *p* < 0.05; g: AUC (MTA‐avg) vs. AUC (HF), *p* < 0.05; h: AUC (MTA‐avg) vs. AUC (ILV volume), *p* < 0.05; i: AUC (MTA‐avg) vs. AUC(ILV fraction), *p* < 0.05; j: AUC(HV) vs. AUC(HF), *p* < 0.05; k: AUC(HV) vs. AUC(ILV volume), *p* < 0.05; l: AUC (HV) vs. AUC (ILV fraction), *p* < 0.05; m: AUC (HF) vs. AUC (ILV volume), *p* < 0.05; n: AUC (HF) vs. AUC (ILV fraction), *p* < 0.05; o: AUC (ILV volume) vs. AUC (ILV fraction), *p* < 0.05.

Abbreviations: AUC, area under the curve; CI, confidence interval; ILV, inferior lateral ventricle; MTA scale, visual rating of medial temporal lobe atrophy (average score of left and right hemispheres); QMTA, automatic quantification of medial temporal lobe atrophy based on total ILV volume divided by total hippocampal volume.

##### NC A− and MCIc A+

The sMRI indices could distinguish amyloid‐positive MCIc and NC (Table 4), and QMTA (AUC = 0.883), with a moderate sensitivity of 77.58% and specificity of 88.00%, had the best diagnostic performance, which was significantly higher than that of MTA, HV, ILV volume, and ILV fraction with statistical differences. Although the diagnostic accuracy of QMTA was higher than that of HF (AUC = 0.849), the difference was not statistically significant (*p* = 0.073).

##### MCIs A− and MCIc A+

Comparing the MCIs A− and MCIc A+ groups (see Table S12), there were statistical differences between MCIc A+ and MCI A− in cognition, hippocampus, ILV, MTA, and QMTA. Distinguishing between MCIs A− and MCIc A+ (Table 4), QMTA had the highest diagnostic accuracy (AUC = 0.766), which was higher than that of MTA, hippocampus, and ILV, and the sensitivity of QMTA was improved (75.76%). Compared with other single brain structure volumetry indices, MTA‐avg was not better in distinguishing MCIs and MCIc.

### 3.6 Identifying cutoff values for AD versus MCI to AD

The accuracy of the QMTA and MTA diagnostic cutoff values in differentiating early AD was analyzed, and their diagnostic value was compared with MTA's Scheltens' criteria (Table 5). The best cutoff value of QMTA was 0.635; its sensitivity and specificity were 92.45% and 90.26%, respectively, with an FPR of 9.74% and FNR of 7.55%. The sensitivity and specificity of age ≤75 years and >75 years were 86.77% and 93.30% as well as 97.12% and 84.09%, respectively. While Scheltens' criteria's sensitivity of ≤75 and >75 years old was 82.01% and 43.88%, the higher specificity was 92.18% and 100.00%, while FPR was 7.82% and 0.00%, and FNR was 17.99% and 56.12%, respectively.

**TABLE 5:** Cutoff values of QMTA and MTA in distinguishing AD from NC.

	Age Group	Cutoffs	Sen (%)	Spec (%)	FPR (%)	FNR (%)	PPV (%)	NPV (%)
QMTA	Total	0.635	92.45	90.26	9.74	7.55	83.17	95.83
	≤75 yrs	0.635	86.77	93.30	6.70	12.23	83.56	95.16
	>75 yrs	0.635	97.12	84.09	15.91	2.88	82.82	97.37
MTA‐avg	Total	1.5	93.19	77.90	22.10	6.81	68.70	95.63
	≤75 yrs	1.5	90.65	82.40	17.60	9.35	66.67	95.78
	>75 yrs	1.5	95.68	68.75	31.25	4.32	70.74	95.28
MTA Scheltens' criteria	Total		62.95	94.76	5.24	37.05	86.21	83.09
	≤75 yrs	2.0	82.01	92.18	7.82	17.99	80.28	92.96
	>75 yrs	3.0	43.88	100.00	0	56.12	100.00	69.29

Abbreviations: AUC, area under the curve; CI, confidence intervals; FNR, false‐negative rate; FPR, false‐positive rate; NPV, negative predictive value; PPV, positive predictive values; Sen, sensitivity; Spec, specificity; yrs, years.

To distinguish the conversion of MCI into AD in the MCI population (see Table S13), the best cutoff value of QMTA was 0.635, with an FPR of 33.51% and an FNR of 32.80%; the best cutoff value of MTA was 1.00, FPR was 87.09% and FNR was 4.50%, which led to many false positive results.

## 4 DISCUSSION

This study analyzed the accuracy of the QMTA and MTA scores in the diagnosis of M‐AD or MCI. The results showed that the diagnostic accuracy of QMTA in distinguishing NC from M‐AD was the highest, with an AUC of 0.976, specificity and sensitivity of more than 90%, and better performance of the ATN subgroup analysis of QMTA's diagnostic accuracy. QMTA achieved the best performance in distinguishing NC from MCI, MCIs, and MCIc. The accuracy of QMTA in distinguishing people who converted from MCI to AD was above 0.700, which was higher than that of the other quantitative indicators. According to the subgroup analysis of different sex and age groups, the accuracy of QMTA in distinguishing the NC, M‐AD, and MCI subgroups was still the best.

QMTA is a quantitative MTA index derived from the volume of the hippocampus and ILV, which showed a strong correlation with MTA and ILV, a moderate correlation with the hippocampus, and stronger correlation than the MTA correlation results, which better confirmed the linear combination conclusion of MTA model construction based on the volume of the hippocampus and ILV.^31^ Both QMTA and MTA were significantly correlated with cognitive assessment; however, the correlation of QMTA was stronger. These results were consistent with those of a previous study.^32^ Both QMTA and MTA were related to CSF biomarkers, indicating the possibility that AD neuropathology begins with changes in the medial temporal lobe.^33^

The MTA scale is an important tool for early AD screening and diagnosis,^34^ but as a qualitative measure based on 2D MR or CT images,^17^ it cannot sensitively identify early or subtle structural changes in AD or MCI.^10^, ^35^ Meanwhile, MTA visual scoring relies entirely on professional raters, which lacks objectivity and efficiency.^36^ Automatic measurement tools based on artificial intelligence have provided standardized and highly reproducible results.^31^, ^37^ QMTA, as a quantitative index based on the measurement of 3D images, can observe subtle and systematic changes in brain structure.^38^

Based on the MTA scale, Scheltens' criteria were often used to assist in distinguishing AD from NC worldwide, which showed a sensitivity of 81.00% and specificity of 67.00% at a cutoff of 2 (≤75 years) or 3 (>75 years).^17^ In this study, we found that QMTA, compared with MTA scale and other indicators, achieved the highest accuracy in distinguishing AD from NC with higher sensitivity and specificity, while adjusting for age, sex, and education level, which did not change this result. We found that the MTA scale could achieve a certain degree of sensitivity in correctly distinguishing AD from NC, but it manifested a misdiagnosis rate as FNR reached 37.05%. QMTA could greatly reduce the misdiagnosis rate with an FNR of 7.55%, at the same time improving the sensitivity to 90.26% at the cutoff of 0.635. And studies from other databases have confirmed that the QMTA has greater diagnostic accuracy than a single brain structural index, such as the hippocampus, to differentiate AD.^14^, ^39^ Thus, it might be more suitable for the clinical diagnosis of AD.

MCI is an important target for preventing the progression of AD to dementia. The definition and diagnosis of MCI were determined by cognitive measurement and clinical diagnosis,^40^ but a single cognitive assessment predicted MCI conversion to AD with low accuracy.^41^ An autopsy study of hippocampal volume based on MRI found that hippocampal volume was more in line with the neuropathology of Alzheimer's disease than with clinical diagnosis or cognitive measurement.^42^ In the present study, we found that the accuracy of QMTA in distinguishing MCI from NC was 0.7. MTA is also considered a marker for diagnosing the conversion of MCI into AD.^16^ To identify patients with MCI who had a higher chance of converting to AD, previous studies combined MRI, CSF, and neurocognitive scale machine learning, and the accuracy of the classification of MCIs and MCIc was reported to be 0.670.^43^ In addition, fusion of MRI, FDG‐PET, and CSF data for machine learning, regression analysis of MMSE and ADAS‐Cog scores, and classification accuracy of MCIs and MCIc was 0.739.^44^ The accuracy of QMTA in this study to distinguish between MCIs and MCIc was as high as 0.725, which is similar to the highest reported multi‐domain classification accuracy.^45^, ^46^ However, as a single indicator only replied on structural MRI, QMTA is more easily accessible, practical, and cost‐effective as a good clinical screening tool.

The MTA assessment ranges from 0 to 4, and the study found that patients with AD or MCI can increase their MTA score by 1 point in an average of 8 years.^35^ In comparison, the rate of hippocampal atrophy varies at different ages, resulting in different diagnostic criteria for MTA around the age of 75.^47^ Age influences MTA diagnosis accuracy.^48^ The present study found that the diagnostic accuracy, sensitivity, and specificity of QMTA in different sexes and ages are similar; thus, it is less affected by age and sex. Amyloid levels can predict the risk of early AD progression.^7^ When the objective was changed to identify AD confirmed by A+ and MCIc confirmed by A+, the quantitative MRI‐based indices, including QMTA, showed higher diagnostic accuracy for distinguishing NC from AD or MCIc and the conversion of MCI to AD. The accuracy, sensitivity, and specificity of QMTA were higher than those of MTA. QMTA may be a quantitative continuous index that can be used to sensitively observe changes in the medial temporal lobe.

The strengths of this study were the multicenter‐based large sample size, use of artificial intelligence automated quantitative MRI analysis tools to quantify brain structure and quantitative MTA metrics, and comparison of quantitative metrics across subgroups to identify early AD accuracy. There were also some limitations to this study. First, the follow‐up time of some MCI patients was too short (only 1 year), and some of them were not converted to AD and listed as MCIs after 1 year of follow‐up, which might have led to the estimation error of sensitivity and specificity of QMTA in distinguishing the conversion of MCI to AD. Another limitation was that there was no clinical symptom grouping of MCI patients, that is, amnestic MCI or non‐amnestic MCI. Amnestic MCI is more likely to be converted to AD; thus, it could be better to analyze them.

## 5 CONCLUSION

QMTA can be used as a reliable and effective tool for assisting in the clinical diagnosis of AD. The diagnostic performance of QMTA in the early identification of AD or MCI was better than that of MTA, the hippocampus, and ILV. Compared with MTA, QMTA is reproducible and objective in estimating the risk of MCI conversion and the onset of AD.

## AUTHOR CONTRIBUTIONS

Dr. Jun Liu and Dr. Shi Lin had full access to all data in the study and were responsible for the integrity of the data and the accuracy of the data analysis. Liu Jun, Shi Lin, Mai Yingren, Cao Zhiyu, and Zhao Lei contributed to the study concept and design. All authors contributed to the acquisition, analysis, or interpretation of data. Mai Yingren and Cao Zhiyu contributed to the drafting of the manuscript. Yingren Mai, Zhiyu Cao, Lei Zhao, Qun Yu, Vincent CT Mok, Lin Shi, and Jun Liu contributed to the critical revision of the manuscript for important intellectual content. Mai Yingren, Cao Zhiyu, and Zhao Lei contributed to the statistical analysis. Liu Jun obtained funding. Liu Jun and Shi Lin contributed to the administrative, technical, or material support, and study supervision.

## FUNDING INFORMATION

This study was supported by the grant provided by funding from the National Nature Science Foundation of China (grant number 82171178), the Natural Science Foundation of Guangdong Province (grant number 2020A1515010210), the Research and Development program in key areas of Science and Technology Program of Guangzhou, China (grant numbers 202007030010).

## CONFLICT OF INTEREST STATEMENT

Lin Shi is the director of BrainNow Medical Technology Limited. Vincent CT Mok is the chief medical consultant of BrainNow Medical Technology Limited. Wenyan Liu, Yishan Luo, and Lei Zhao are now employed by BrainNow Medical Technology Limited. All other authors report no financial relationships with commercial interests.

## Supporting information


Tables S1‐S13

## Data Availability

Data are available in a public, open‐access repository. All ADNI data are deposited in a publicly accessible repository and can be accessed at http://adni.loni.usc.edu.
