# Supplementary material for: The role of visual rating and automated brain volumetry in early detection and differential diagnosis of Alzheimer's disease
Source: CNS Neurosci Ther. 2023 Oct 21;30(4):e14492. doi: 10.1111/cns.14492 (PMC11017425; doi:10.1111/cns.14492)
Supplement: Supplementary file 1 — Tables S1‐S13 [file CNS-30-e14492-s001.docx]

**Supplementary Online Content**

**eTable 1.** Single measure and average measure ICC results for MTA

|  | Single-Measures ICC | | Average-Measures ICC | |
| --- | --- | --- | --- | --- |
| Scale | 4 raters, n = 100 | 2 raters, n = 100 | 4 raters, n = 100 | 2 raters, n = 100 |
| MTA-L | 0.720 (0.645, 0.787) | 0.911 (0.870, 0.939) | 0.911 (0.879, 0.937) | 0.953 (0.931, 0.969) |
| MTA-R | 0.813 (0.757, 0.861) | 0.914 (0.875, 0.941) | 0.946 (0.926, 0.961) | 0.955 (0.933, 0.970) |
| MTA-avg | 0.821 (0.768, 0.867) | 0.908 (0.867, 0.937) | 0.948 (0.930, 0.963) | 0.952 (0.929, 0.968) |
|  | Single-Measures ICC | | Average-Measures ICC | |
| Scale | 2 raters, n = 1696 | | 2 raters, n = 1696 | |
| MTA-L | 0.894 (0.877, 0.908) | | 0.944 (0.935, 0.952) | |
| MTA-R | 0.895 (0.878, 0.909) | | 0.944 (0.935, 0.952) | |
| MTA-avg | 0.885 (0.866, 0.900) | | 0.939 (0.928, 0.947) | |

MTA: visual rating of medial temporal lobe atrophy; ICC: intraclass correlation coefficient; L: left; R: right; avg: average.

**eTable 2.** Demographic characteristics, brain structure, and performance across ATN subgroups

|  | N | NC (n=325) | MCIs (n=425) | MCIc (n=214) | AD (n=167) |
| --- | --- | --- | --- | --- | --- |
| Age (years), median(IQR) | 1131 | 72.00 (68.00, 77.00) | 72.00 (66.00, 77.00) | 74.00 (69.00, 78.70) | 75.00 (69.00, 79.20)† |
| Male, n (%) | 1131 | 136 (41.84) | 235 (55.29) * | 133 (62.15) *† | 105 (62.87) *†‡ |
| Education (years), median(IQR) | 1131 | 16.00 (15.00, 18.00) | 16.00 (14.00, 18.00) | 16.00 (14.00, 18.00) | 16.00 (13.00, 18.00)* |
| MMSE, median(IQR) | 1131 | 29.00 (29.00, 30.00) | 29.00 (27.00, 29.00)* | 27.00 (26.00, 30.00)*† | 23.00 (22.00, 25.00)*†‡ |
| FAQ, median(IQR) | 1121 | 0.00 (0.00, 0.00) | 1.00 (0.00, 3.00)* | 4.00 (1.00, 7.00)*† | 12.00 (9.00, 18.00)*†‡ |
| CDR, median(IQR) | 1131 | 0.00 (0.00, 0.00) | 0.50 (0.50, 0.50)* | 0.50 (0.50, 0.50)* | 1.00 (0.50, 1.00)*†‡ |
| CDR-SOB, median(IQR) | 1131 | 0.00 (0.00, 0.00) | 1.00 (0.50, 1.50)* | 1.50 (1.00, 2.50)* † | 4.50 (3.50, 5.00)*†‡ |
| ADAS-cog, median(IQR) | 1131 | 7.00 (4.00, 9.33) | 9.00 (6.00, 13.00)* | 16.00 (11.00, 20.75)*† | 24.00 (18.00, 30.00)*†‡ |
| CSF Aβ_42_ (pg/ml), median(IQR) | 1131 | 1529.00 (1182.00, 2022.00) | 1077.00 (736.50, 1633.50)* | 669.90 (553.00, 863.25)*† | 600.00 (462.00, 749.00)*† |
| CSF P-tau^181^ (pg/ml), median(IQR) | 1122 | 19.00 (15.43, 24.79) | 20.80 (15.93, 28.52)* | 32.00 (24.43, 46.42)*† | 32.70 (26.36, 40.96)*† |
| CSF T-tau (pg/ml),median(IQR) | 1122 | 215.45 (176.90, 270.95) | 233.60 (180.50, 302.75) | 326.00 (252.00, 434.10)* | 325.60 (271.10, 400.55)*† |
| HV (ml), mean ± SD | 1131 | 6.36 ± 0.74 | 6.05 ± 0.89* | 5.50 ± 0.93*† | 5.05 ± 0.93*†‡ |
| HF, mean ± SD | 1131 | 0.433 ± 0.046 | 0.405 ± 0.060* | 0.364 ± 0.058*† | 0.333 ± 0.057*†‡ |
| ILV volume(ml), median(IQR) | 1131 | 2.73 (2.33, 3.23) | 3.19 (2.54, 4.05)* | 4.03 (3.07, 5.21)*† | 5.05 (4.13, 6.49)*†‡ |
| ILV fraction, median(IQR) | 1131 | 0.192 (0.163, 0.211) | 0.212 (0.174, 0.257)* | 0.265 (0.204, 0.329)*† | 0.341 (0.269, 0.409)*†‡ |
| QMTA, median(IQR) | 1131 | 0.432 (0.373, 0.503) | 0.516 (0.403, 0.695)* | 0.715 (0.560, 0.974)*† | 1.060 (0.782, 1.320)*†‡ |
| MTA-avg,median(IQR) | 1131 | 1.00 (0.50, 1.00) | 1.00 (1.00, 2.00)* | 2.00 (1.00, 2.50)*† | 2.50 (2.00, 3.00)*†‡ |

IQR: interquartile rang; FAQ: Functional Activities Questionnaire; CDR: Clinical Dementia Rating; CDR-SOB: Clinical Dementia Rating sum of boxes; ADAS-cog: Alzheimer’s disease assessment scale-cognitive; HV: the absolute volumes of hippocampus; HF: Hippocampal fraction; ILV: Inferior lateral ventricle; avg: average; * With significant difference from NC (*P*<0.05); † With significant difference from MCIs (*P* <0.05)；‡ With significant difference from MCIc (*P*<0.05).

**eTable 3.** Results of Delong's test of brain structure index in different diagnostic groups

|  | *P1* | *P2* | *P3* | *P4* | *P5* | *P6* | *P7* | *P8* | *P9* | *P10* | *P11* | *P12* | *P13* | *P14* | *P15* |
| --- | --- | --- | --- | --- | --- | --- | --- | --- | --- | --- | --- | --- | --- | --- | --- |
| M-AD vs NC | <0.001 | <0.001 | <0.001 | <0.001 | <0.001 | <0.001 | 0.160 | 0.371 | 0.169 | <0.001 | 0.004 | <0.001 | 0.478 | 0.021 | <0.001 |
| M-AD vs MCI | <0.001 | <0.001 | <0.001 | <0.001 | <0.001 | 0.002 | 0.006 | 0.316 | 0.1270 | 0.317 | 0.047 | <0.001 | 0.076 | <0.001 | <0.001 |
| M-AD vs MCIs | <0.001 | <0.001 | <0.001 | <0.001 | <0.001 | 0.003 | 0.022 | 0.283 | 0.117 | 0.188 | 0.066 | <0.001 | 0.182 | 0.002 | <0.001 |
| M-AD vs MCIc | 0.025 | <0.001 | <0.001 | <0.001 | 0.165 | 0.001 | 0.007 | 0.450 | 0.285 | 0.763 | 0.090 | 0.005 | 0.063 | 0.004 | <0.001 |
| MCI vs NC | 0.004 | <0.001 | 0.113 | <0.001 | <0.001 | 0.035 | 0.488 | 0.304 | 0.363 | <0.001 | 0.270 | 0.193 | 0.156 | 0.229 | 0.699 |
| MCIs vs NC | 0.246 | 0.003 | 0.292 | <0.001 | <0.001 | 0.036 | 0.953 | 0.429 | 0.344 | 0.005 | 0.220 | 0.208 | 0.594 | 0.573 | 0.816 |
| MCIc vs NC | <0.001 | <0.001 | 0.054 | <0.001 | <0.001 | 0.264 | 0.056 | 0.306 | 0.683 | <0.001 | 0.728 | 0.445 | 0.016 | 0.053 | 0.178 |
| MCIc vs MCIs | <0.001 | 0.081 | 0.307 | <0.001 | <0.001 | 0.670 | 0.140 | 0.632 | 0.767 | 0.161 | 0.552 | 0.828 | 0.111 | 0.277 | 0.095 |

*P1*: QMTA vs MTA; *P2*: QMTA vs HV; *P3*: QMTA vs HF; *P4*: QMTA vs ILV volume; *P5*: QMTA vs ILV fraction; *P6*: MTA-avg vs HV; *P7*: MTA-avg vs HF; *P8:* MTA-avg vs ILV volume; *P9*: MTA-avg vs ILV fraction; *P10*: HV vs HF; *P11*: HV vs ILV volume; *P12:* HV vs ILV fraction; *P13*: HF vs ILV volume; *P14*: HV vs ILV fraction; *P15:* ILV volume vs ILV fraction.

**eTable 4.** AUC value after adjusting for covariates of age, gender, and education level

|  | Mean | SD | Standard Error | *P-*value |
| --- | --- | --- | --- | --- |
| QMTA |  |  |  |  |
| Age | -0.0021 | 0.0101 | 0.0036 | 0.580 |
| Gender | 0.0022 | 0.0088 | 0.0032 | 0.509 |
| Education | -0.0021 | 0.0023 | 0.0008 | 0.040 |
| MTA-avg |  |  |  |  |
| Age | -0.0037 | 0.0076 | 0.0027 | 0.205 |
| Gender | -0.0023 | 0.0028 | 0.0010 | 0.054 |
| Education | -0.0037 | 0.0046 | 0.0016 | 0.059 |
| Hippocampal volume (ml) |  |  |  |  |
| Age | 0.0050 | 0.0059 | 0.0021 | 0.049 |
| Gender | -0.0206 | 0.0112 | 0.0039 | 0.001 |
| Education | 0.0077 | 0.0041 | 0.0014 | 0.001 |
| Hippocampal fraction (%) |  |  |  |  |
| Age | 0.0033 | 0.0040 | 0.0014 | 0.055 |
| Gender | 0.0050 | 0.0070 | 0.0025 | 0.085 |
| Education | -0.0009 | 0.0017 | 0.0006 | 0.168 |
| ILV volume (ml) |  |  |  |  |
| Age | 0.0008 | 0.0085 | 0.0030 | 0.805 |
| Gender | -0.0039 | 0.0175 | 0.0062 | 0.551 |
| Education | -0.0097 | 0.0059 | 0.0021 | 0.002 |
| ILV fraction (%) |  |  |  |  |
| Age | -0.0013 | 0.0088 | 0.0031 | 0.694 |
| Gender | 0.0020 | 0.0059 | 0.0021 | 0.370 |
| Education | -0.0039 | 0.0036 | 0.0013 | 0.018 |

ILV: Inferior lateral ventricle; avg: average.

**etable 5.** Correct of AUC values to distinguish M-AD

|  | QMTA | MTA-avg | Hippocampal volume (ml) | Hippocampal fraction (%) | ILV volume (ml) | ILV fraction (%) |
| --- | --- | --- | --- | --- | --- | --- |
| **M-AD vs NC** | 0.976 | 0.937 | 0.881 | 0.920 | 0.928 | 0.947 |
| age | 0.975 | 0.938 | 0.876 | 0.915 | 0.924 | 0.941 |
| gender | 0.973 | 0.938 | 0.901 | 0.912 | 0.932 | 0.941 |
| education | 0.974 | 0.935 | 0.863 | 0.917 | 0.939 | 0.948 |
| age & gender | 0.976 | 0.934 | 0.897 | 0.910 | 0.927 | 0.939 |
| age & education | 0.975 | 0.937 | 0.859 | 0.912 | 0.942 | 0.948 |
| gender & education | 0.974 | 0.934 | 0.889 | 0.910 | 0.939 | 0.947 |
| age & gender & education | 0.976 | 0.935 | 0.888 | 0.904 | 0.939 | 0.945 |
| **M-AD vs MCI** | 0.836 | 0.799 | 0.743 | 0.755 | 0.787 | 0.815 |
| age | 0.830 | 0.797 | 0.730 | 0.749 | 0.778 | 0.806 |
| gender | 0.838 | 0.801 | 0.753 | 0.756 | 0.806 | 0.817 |
| education | 0.836 | 0.802 | 0.729 | 0.751 | 0.797 | 0.815 |
| age & gender | 0.837 | 0.798 | 0.746 | 0.748 | 0.800 | 0.811 |
| age & education | 0.834 | 0.799 | 0.722 | 0.748 | 0.792 | 0.811 |
| gender & education | 0.837 | 0.804 | 0.743 | 0.751 | 0.811 | 0.818 |
| age & gender & education | 0.839 | 0.798 | 0.737 | 0.741 | 0.806 | 0.813 |
| **M-AD vs MCIs** | 0.894 | 0.853 | 0.801 | 0.817 | 0.840 | 0.868 |
| age | 0.889 | 0.848 | 0.787 | 0.806 | 0.831 | 0.861 |
| gender | 0.896 | 0.856 | 0.809 | 0.812 | 0.856 | 0.867 |
| education | 0.889 | 0.849 | 0.792 | 0.814 | 0.845 | 0.865 |
| age & gender | 0.896 | 0.852 | 0.803 | 0.811 | 0.853 | 0.863 |
| age & education | 0.892 | 0.848 | 0.779 | 0.805 | 0.841 | 0.861 |
| gender & education | 0.895 | 0.852 | 0.802 | 0.810 | 0.857 | 0.866 |
| age & gender & education | 0.896 | 0.851 | 0.799 | 0.808 | 0.855 | 0.862 |
| **M-AD vs MCIc** | 0.730 | 0.701 | 0.637 | 0.642 | 0.690 | 0.717 |
| age | 0.716 | 0.703 | 0.624 | 0.637 | 0.681 | 0.712 |
| gender | 0.733 | 0.705 | 0.638 | 0.635 | 0.710 | 0.721 |
| education | 0.734 | 0.708 | 0.627 | 0.640 | 0.702 | 0.725 |
| age & gender | 0.729 | 0.700 | 0.637 | 0.637 | 0.708 | 0.715 |
| age & education | 0.729 | 0.709 | 0.614 | 0.635 | 0.702 | 0.720 |
| gender & education | 0.735 | 0.711 | 0.633 | 0.640 | 0.719 | 0.720 |
| age & gender & education | 0.730 | 0.710 | 0.632 | 0.636 | 0.712 | 0.720 |

ILV, inferior lateral ventricle; MTA: visual rating of medial temporal lobe atrophy (average score of left and right hemispheres); vs: versus.

**etable 6.** Correct of AUC values to distinguish MCI

|  | QMTA | MTA-avg | Hippocampal volume (ml) | Hippocampal fraction (%) | ILV volume (ml) | ILV fraction (%) |
| --- | --- | --- | --- | --- | --- | --- |
| **MCI vs NC** | 0.734 | 0.703 | 0.668 | 0.714 | 0.691 | 0.693 |
| age | 0.746 | 0.713 | 0.667 | 0.714 | 0.695 | 0.700 |
| gender | 0.719 | 0.701 | 0.696 | 0.699 | 0.673 | 0.678 |
| education | 0.733 | 0.709 | 0.663 | 0.714 | 0.694 | 0.691 |
| age & gender | 0.735 | 0.706 | 0.699 | 0.701 | 0.678 | 0.687 |
| age & education | 0.747 | 0.715 | 0.663 | 0.719 | 0.703 | 0.702 |
| gender & education | 0.722 | 0.702 | 0.693 | 0.699 | 0.677 | 0.680 |
| age & gender & education | 0.736 | 0.709 | 0.697 | 0.702 | 0.687 | 0.691 |
| **MCIs vs NC** | 0.662 | 0.646 | 0.605 | 0.645 | 0.635 | 0.633 |
| age | 0.679 | 0.659 | 0.606 | 0.646 | 0.646 | 0.646 |
| gender | 0.646 | 0.642 | 0.630 | 0.625 | 0.613 | 0.623 |
| education | 0.661 | 0.650 | 0.595 | 0.643 | 0.636 | 0.632 |
| age & gender | 0.666 | 0.653 | 0.636 | 0.631 | 0.627 | 0.637 |
| age & education | 0.679 | 0.660 | 0.601 | 0.650 | 0.649 | 0.650 |
| gender & education | 0.646 | 0.646 | 0.627 | 0.628 | 0.618 | 0.621 |
| age & gender & education | 0.668 | 0.655 | 0.634 | 0.633 | 0.625 | 0.637 |
| **MCIc vs NC** | 0.866 | 0.808 | 0.786 | 0.840 | 0.794 | 0.803 |
| age | 0.866 | 0.807 | 0.779 | 0.835 | 0.788 | 0.802 |
| gender | 0.854 | 0.804 | 0.823 | 0.830 | 0.777 | 0.788 |
| education | 0.868 | 0.812 | 0.782 | 0.841 | 0.800 | 0.802 |
| age & gender | 0.862 | 0.795 | 0.822 | 0.829 | 0.775 | 0.786 |
| age & education | 0.872 | 0.812 | 0.778 | 0.839 | 0.801 | 0.805 |
| gender & education | 0.861 | 0.802 | 0.822 | 0.833 | 0.783 | 0.788 |
| age & gender & education | 0.865 | 0.801 | 0.823 | 0.834 | 0.786 | 0.792 |
| **MCIc vs MCIs** | 0.725 | 0.682 | 0.691 | 0.709 | 0.675 | 0.686 |
| age | 0.716 | 0.673 | 0.687 | 0.701 | 0.664 | 0.676 |
| gender | 0.724 | 0.680 | 0.712 | 0.705 | 0.678 | 0.682 |
| education | 0.723 | 0.673 | 0.685 | 0.706 | 0.677 | 0.685 |
| age & gender | 0.718 | 0.677 | 0.699 | 0.701 | 0.671 | 0.673 |
| age & education | 0.714 | 0.671 | 0.683 | 0.702 | 0.665 | 0.675 |
| gender & education | 0.722 | 0.679 | 0.708 | 0.708 | 0.678 | 0.683 |
| age & gender & education | 0.720 | 0.674 | 0.700 | 0.7012 | 0.673 | 0.674 |

ILV, inferior lateral ventricle; MTA: visual rating of medial temporal lobe atrophy (average score of left and right hemispheres); vs: versus.

**eTable 7.** ROC curve analyses for differentiating different diagnoses with SMRI indexes on the ≤75 years old

|  | AUC (95% CI) | Youden index (%) | Optimal threshold | Sensitivity (%) | Specificity (%) |
| --- | --- | --- | --- | --- | --- |
| **QMTA** |  |  |  |  |  |
| M-AD vs NC (n=497) | 0.979 (0.962, 0.990) | 85.11 | ≥0.562 | 97.12 | 87.99 |
| M-AD vs MCI (n=684) | 0.853 (0.825, 0.879) | 55.53 | ≥0.675 | 84.89 | 70.64 |
| M-AD vs MCIs (n=505) | 0.908 (0.879, 0.931) | 66.04 | ≥0.675 | 84.89 | 81.15 |
| M-AD vs MCIc (n=318) | 0.743 (0.691, 0.790) | 35.00 | ≥0.721 | 79.14 | 55.87 |
| MCI vs NC (n=903) | 0.705 (0.674, 0.734) | 33.38 | ≥0.555 | 46.79 | 86.59 |
| MCIs vs NC (n=724) | 0.632 (0.596, 0.667) | 22.18 | ≥0.504 | 45.08 | 77.09 |
| MCIc vs NC (n=537) | 0.853 (0.820, 0.882) | 57.54 | ≥0.552 | 71.51 | 86.03 |
| MCIc vs MCIs (n=545) | 0.734 (0.695, 0.771) | 36.56 | ≥0.548 | 72.63 | 63.93 |
| **MTA-scale (average)** |  |  |  |  |  |
| M-AD vs NC (n=497) | 0.931 (0.905, 0.951) | 74.19 | ≥1.50 | 82.01 | 92.18 |
| M-AD vs MCI (n=684) | 0.800 (0.768, 0.829) | 49.90 | ≥1.50 | 82.01 | 67.89 |
| M-AD vs MCIs (n=505) | 0.853 (0.819, 0.883) | 58.24 | ≥1.50 | 82.01 | 76.23 |
| M-AD vs MCIc (n=318) | 0.692 (0.638, 0.742) | 32.85 | ≥1.50 | 82.01 | 50.84 |
| MCI vs NC (n=903) | 0.691 (0.660, 0.721) | 28.82 | ≥1.00 | 46.42 | 82.40 |
| MCIs vs NC (n=724) | 0.637 (0.600, 0.672) | 19.01 | ≥1.00 | 36.61 | 82.40 |
| MCIc vs NC (n=537) | 0.802 (0.766, 0.835) | 48.88 | ≥1.00 | 66.48 | 82.40 |
| MCIc vs MCIs (n=545) | 0.687 (0.646, 0.726) | 29.87 | ≥1.00 | 66.48 | 63.39 |
| **Hippocampal volume (ml)** |  |  |  |  |  |
| M-AD vs NC (n=497) | 0.879 (0.847, 0.906) | 62.42 | ≤5.78 | 80.58 | 81.84 |
| M-AD vs MCI (n=684) | 0.765 (0.732, 0.797) | 40.61 | ≤5.44 | 69.78 | 70.83 |
| M-AD vs MCIs (n=505) | 0.812 (0.775, 0.845) | 50.33 | ≤5.56 | 74.10 | 76.23 |
| M-AD vs MCIc (n=318) | 0.670 (0.615, 0.721) | 27.93 | ≤4.83 | 47.48 | 80.45 |
| MCI vs NC (n=903) | 0.647 (0.614, 0.678) | 23.68 | ≤5.77 | 41.83 | 81.84 |
| MCIs vs NC (n=724) | 0.590 (0.553 0.626) | 14.20 | ≤6.21 | 52.19 | 62.01 |
| MCIc vs NC (n=537) | 0.763 (0.725, 0.798) | 46.09 | ≤5.77 | 64.25 | 81.84 |
| MCIc vs MCIs (n=545) | 0.681 (0.640, 0.720) | 34.06 | ≤5.58 | 58.10 | 75.96 |
| **Hippocampal fraction (%)** |  |  |  |  |  |
| M-AD vs NC (n=497) | 0.918 (0.890, 0.941) | 68.01 | ≤0.392 | 85.61 | 82.40 |
| M-AD vs MCI (n=684) | 0.770 (0.737, 0.801) | 40.90 | ≤0.369 | 73.38 | 67.52 |
| M-AD vs MCIs (n=505) | 0.826 (0.790, 0.858) | 50.43 | ≤0.369 | 73.38 | 77.05 |
| M-AD vs MCIc (n=318) | 0.656 (0.601, 0.708) | 25.39 | ≤0.341 | 56.12 | 69.27 |
| MCI vs NC (n=903) | 0.689 (0.658, 0.719) | 30.30 | ≤0.411 | 59.63 | 70.67 |
| MCIs vs NC (n=724) | 0.626 (0.590, 0.662) | 20.90 | ≤0.414 | 52.19 | 68.72 |
| MCIc vs NC (n=537) | 0.818 (0.782, 0.850) | 51.40 | ≤0.399 | 72.63 | 78.77 |
| MCIc vs MCIs (n=545) | 0.701 (0.661, 0.740) | 32.19 | ≤0.399 | 72.63 | 59.56 |
| **ILV volume (ml)** |  |  |  |  |  |
| M-AD vs NC (n=497) | 0.920 (0.892, 0.942) | 69.12 | ≥3.68 | 80.58 | 88.55 |
| M-AD vs MCI (n=684) | 0.791 (0.758, 0.821) | 45.46 | ≥3.90 | 74.82 | 70.64 |
| M-AD vs MCIs (n=505) | 0.844 (0.810, 0.875) | 55.42 | ≥3.90 | 74.82 | 80.60 |
| M-AD vs MCIc (n=318) | 0.681 (0.627, 0.732) | 26.19 | ≥4.51 | 59.71 | 66.48 |
| MCI vs NC (n=903) | 0.663 (0.631, 0.694) | 26.27 | ≥3.44 | 42.75 | 83.52 |
| MCIs vs NC (n=724) | 0.606 (0.570, 0.642) | 18.45 | ≥3.40 | 36.89 | 81.56 |
| MCIc vs NC (n=537) | 0.779 (0.742, 0.814) | 44.41 | ≥3.50 | 59.78 | 84.64 |
| MCIc vs MCIs (n=545) | 0.686 (0.645, 0.724) | 32.31 | ≥3.84 | 53.07 | 79.23 |
| **ILV fraction (%)** |  |  |  |  |  |
| M-AD vs NC (n=497) | 0.948 (0.924, 0.966) | 73.80 | ≥0.232 | 87.77 | 86.03 |
| M-AD vs MCI (n=684) | 0.832 (0.802, 0.859) | 52.19 | ≥0.263 | 77.70 | 74.50 |
| M-AD vs MCIs (n=505) | 0.883 (0.852, 0.910) | 61.85 | ≥0.263 | 77.70 | 84.15 |
| M-AD vs MCIc (n=318) | 0.726 (0.674, 0.775) | 32.45 | ≥0.263 | 77.70 | 54.75 |
| MCI vs NC (n=903) | 0.670 (0.639, 0.701) | 27.85 | ≥0.198 | 61.65 | 66.20 |
| MCIs vs NC (n=724) | 0.611 (0.574, 0.647) | 20.57 | ≥0.198 | 54.37 | 66.20 |
| MCIc vs NC (n=537) | 0.791 (0.755, 0.825) | 46.37 | ≥0.216 | 68.16 | 78.21 |
| MCIc vs MCIs (n=545) | 0.695 (0.655, 0.734) | 32.87 | ≥0.246 | 53.63 | 79.23 |

AUC: area under the curve; CI: confidence interval; ILV, inferior lateral ventricle; MTA-scale: visual rating of medial temporal lobe atrophy (average score of left and right hemispheres); vs: versus.

**eTable 8.** ROC curve analyses for differentiating different diagnoses with SMRI indexes on the >75 years old

|  | AUC (95% CI) | Youden index (%) | Optimal threshold | Sensitivity (%) | Specificity (%) |
| --- | --- | --- | --- | --- | --- |
| **QMTA** |  |  |  |  |  |
| M-AD vs NC (n=315) | 0.973 (0.948, 0.988) | 83.98 | ≥0.715 | 91.37 | 92.61 |
| M-AD vs MCI (n=478) | 0.809 (0.771, 0.844) | 48.13 | ≥0.830 | 80.58 | 67.55 |
| M-AD vs MCIs (n=346) | 0.874 (0.834, 0.907) | 58.35 | ≥0.830 | 80.58 | 77.78 |
| M-AD vs MCIc (n=271) | 0.708 (0.650, 0.762) | 32.81 | ≥0.812 | 81.29 | 51.52 |
| MCI vs NC (n=515) | 0.784 (0.746, 0.819) | 46.58 | ≥0.635 | 61.36 | 85.23 |
| MCIs vs NC (n=383) | 0.720 (0.672, 0.764) | 36.50 | ≥0.555 | 63.77 | 72.73 |
| MCIc vs NC (n=308) | 0.885 (0.845, 0.919) | 64.77 | ≥0.635 | 79.55 | 85.23 |
| MCIc vs MCIs (n=339) | 0.707 (0.655, 0.755) | 30.13 | ≥0.855 | 48.48 | 81.64 |
| **MTA-scale (average)** |  |  |  |  |  |
| M-AD vs NC (n=315) | 0.938 (0.905, 0.962) | 72.47 | ≥1.50 | 90.65 | 81.82 |
| M-AD vs MCI (n=478) | 0.788 (0.749, 0.824) | 44.45 | ≥2.00 | 68.35 | 76.11 |
| M-AD vs MCIs (n=346) | 0.841 (0.798, 0.878) | 51.92 | ≥2.00 | 68.35 | 83.57 |
| M-AD vs MCIc (n=271) | 0.705 (0.647, 0.759) | 32.74 | ≥2.00 | 68.35 | 64.39 |
| MCI vs NC (n=515) | 0.719 (0.678, 0.758) | 33.06 | ≥1.00 | 64.31 | 68.75 |
| MCIs vs NC (n=383) | 0.663 (0.613, 0.710) | 23.82 | ≥1.00 | 55.07 | 68.75 |
| MCIc vs NC (n=308) | 0.808 (0.759, 0.850) | 47.54 | ≥1.00 | 78.79 | 68.75 |
| MCIc vs MCIs (n=339) | 0.664 (0.611, 0.714) | 24.57 | ≥1.50 | 65.15 | 59.42 |
| **Hippocampal volume (ml)** |  |  |  |  |  |
| M-AD vs NC (n=315) | 0.875 (0.833, 0.909) | 59.25 | ≤5.31 | 68.35 | 90.91 |
| M-AD vs MCI (n=478) | 0.698 (0.655, 0.739) | 33.39 | ≤5.14 | 62.59 | 70.80 |
| M-AD vs MCIs (n=346) | 0.770 (0.721, 0.813) | 45.18 | ≤5.21 | 65.47 | 79.71 |
| M-AD vs MCIc (n=271) | 0.587 (0.526, 0.646) | 17.14 | ≤5.14 | 62.59 | 54.55 |
| MCI vs NC (n=515) | 0.702 (0.660, 0.741) | 32.99 | ≤5.93 | 62.54 | 70.45 |
| MCIs vs NC (n=383) | 0.631 (0.581, 0.680) | 24.08 | ≤5.93 | 53.62 | 70.45 |
| MCIc vs NC (n=308) | 0.813 (0.765, 0.855) | 50.57 | ≤5.64 | 70.45 | 80.11 |
| MCIc vs MCIs (n=339) | 0.697 (0.645, 0.746) | 33.97 | ≤5.36 | 59.09 | 74.88 |
| **Hippocampal fraction (%)** |  |  |  |  |  |
| M-AD vs NC (n=315) | 0.918 (0.882, 0.946) | 71.60 | ≤0.367 | 78.42 | 93.18 |
| M-AD vs MCI (n=478) | 0.726 (0.683, 0.765) | 37.39 | ≤0.354 | 73.38 | 64.01 |
| M-AD vs MCIs (n=346) | 0.793 (0.747, 0.835) | 49.21 | ≤0.363 | 76.26 | 72.95 |
| M-AD vs MCIc (n=271) | 0.620 (0.559, 0.678) | 21.65 | ≤0.320 | 48.92 | 72.73 |
| MCI vs NC (n=515) | 0.753 (0.714, 0.790) | 38.27 | ≤0.414 | 74.63 | 63.64 |
| MCIs vs NC (n=383) | 0.678 (0.629, 0.725) | 28.85 | ≤0.413 | 65.22 | 63.64 |
| MCIc vs NC (n=308) | 0.871 (0.828, 0.906) | 59.09 | ≤0.369 | 68.18 | 90.91 |
| MCIc vs MCIs (n=339) | 0.712 (0.661, 0.760) | 36.78 | ≤0.369 | 68.18 | 68.60 |
| **ILV volume (ml)** |  |  |  |  |  |
| M-AD vs NC (n=315) | 0.931 (0.897, 0.956) | 69.74 | ≥4.33 | 77.70 | 92.05 |
| M-AD vs MCI (n=478) | 0.771 (0.730, 0.808) | 42.00 | ≥4.30 | 77.70 | 64.31 |
| M-AD vs MCIs (n=346) | 0.822 (0.777, 0.861) | 50.64 | ≥4.30 | 77.70 | 72.95 |
| M-AD vs MCIc (n=271) | 0.691 (0.632, 0.745) | 28.88 | ≥5.31 | 55.40 | 73.48 |
| MCI vs NC (n=515) | 0.732 (0.692, 0.770) | 37.64 | ≥3.72 | 57.52 | 80.11 |
| MCIs vs NC (n=383) | 0.686 (0.637, 0.732) | 32.86 | ≥3.73 | 52.17 | 80.68 |
| MCIc vs NC (n=308) | 0.805 (0.756, 0.847) | 46.02 | ≥3.72 | 65.91 | 80.11 |
| MCIc vs MCIs (n=339) | 0.646 (0.593, 0.697) | 23.17 | ≥4.61 | 43.94 | 79.23 |
| **ILV fraction (%)** |  |  |  |  |  |
| M-AD vs NC (n=315) | 0.941 (0.909, 0.965) | 71.45 | ≥0.290 | 77.70 | 93.75 |
| M-AD vs MCI (n=478) | 0.785 (0.746, 0.821) | 43.02 | ≥0.289 | 78.42 | 64.60 |
| M-AD vs MCIs (n=346) | 0.841 (0.798, 0.878) | 50.40 | ≥0.289 | 78.42 | 71.98 |
| M-AD vs MCIc (n=271) | 0.699 (0.640, 0.753) | 31.45 | ≥0.289 | 78.42 | 53.03 |
| MCI vs NC (n=515) | 0.728 (0.687, 0.766) | 35.18 | ≥0.243 | 60.18 | 75.00 |
| MCIs vs NC (n=383) | 0.674 (0.625, 0.721) | 30.28 | ≥0.211 | 70.05 | 60.23 |
| MCIc vs NC (n=308) | 0.811 (0.763, 0.854) | 49.24 | ≥0.243 | 74.24 | 75.00 |
| MCIc vs MCIs (n=339) | 0.662 (0.609, 0.712) | 26.23 | ≥0.259 | 64.39 | 61.84 |

AUC: area under the curve; CI: confidence interval; ILV, inferior lateral ventricle; MTA-scale: visual rating of medial temporal lobe atrophy (average score of left and right hemispheres); vs: versus.

**eTable 9.** Demographic characteristics and brain structure across gender subgroups

|  | Male (n=900) | Female (n=796) | *P* |
| --- | --- | --- | --- |
| Age (years), median(IQR) | 74.00 (69.00, 79.00) | 73.00 (68.00, 78.00) | 0.019 |
| Education (years), median(IQR) | 16.00 (15.00, 18.00) | 16.00 (13.00, 18.00) | <0.001 |
| HV (ml), mean ± SD | 6.03 ± 1.00 | 5.66 ± 0.95 | <0.001 |
| HF, mean ± SD | 0.377 ± 0.063 | 0.409 ± 0.065 | <0.001 |
| ILV volume(ml), median(IQR) | 3.89 (3.14, 5.10) | 2.82 (2.33, 3.65) | <0.001 |
| ILV fraction, median(IQR) | 0.245 (0.197, 0.322) | 0.204 (0.170, 0.265) | <0.001 |
| QMTA, median(IQR) | 0.642 (0.484, 0.926) | 0.484 (0.383, 0.698) | <0.001 |
| MTA-avg,median(IQR) | 1.50 (1.00, 2.00) | 1.00 (1.00, 2.00) | <0.001 |

IQR: interquartile rang; HV: the absolute volumes of hippocampus; HF: Hippocampal fraction; ILV: Inferior lateral ventricle; avg: average.

**eTable 10.** ROC curve analyses for differentiating different diagnoses with SMRI indexes on the females subgroup

|  | AUC (95% CI) | Youden index (%) | Optimal threshold | Sensitivity (%) | Specificity (%) |
| --- | --- | --- | --- | --- | --- |
| **QMTA** |  |  |  |  |  |
| M-AD vs NC (n=421) | 0.978 (0.959, 0.990) | 86.06 | ≥0.563 | 96.52 | 89.54 |
| M-AD vs MCI (n=490) | 0.855 (0.820, 0.885) | 55.78 | ≥0.596 | 93.91 | 61.87 |
| M-AD vs MCIs (n=369) | 0.902 (0.867, 0.930) | 66.83 | ≥0.589 | 94.78 | 72.05 |
| M-AD vs MCIc (n=236) | 0.756 (0.696, 0.809) | 39.59 | ≥0.684 | 81.74 | 57.85 |
| MCI vs NC (n=681) | 0.695 (0.659, 0.730) | 32.85 | ≥0.547 | 45.60 | 87.25 |
| MCIs vs NC (n=560) | 0.622 (0.580, 0.662) | 21.51 | ≥0.547 | 34.25 | 87.25 |
| MCIc vs NC (n=427) | 0.849 (0.811, 0.881) | 57.00 | ≥0.548 | 69.42 | 87.58 |
| MCIc vs MCIs (n=375) | 0.726 (0.677, 0.770) | 38.51 | ≥0.482 | 81.82 | 56.69 |
| **MTA-scale (average)** |  |  |  |  |  |
| M-AD vs NC (n=421) | 0.933 (0.905, 0.955) | 76.39 | ≥1.50 | 85.22 | 91.18 |
| M-AD vs MCI (n=490) | 0.832 (0.796, 0.864) | 54.82 | ≥1.50 | 85.22 | 69.60 |
| M-AD vs MCIs (n=369) | 0.874 (0.835, 0.906) | 62.78 | ≥1.50 | 85.22 | 77.56 |
| M-AD vs MCIc (n=236) | 0.744 (0.684, 0.799) | 38.11 | ≥1.50 | 85.22 | 52.89 |
| MCI vs NC (n=681) | 0.663 (0.626, 0.699) | 23.21 | ≥1.00 | 43.47 | 79.74 |
| MCIs vs NC (n=560) | 0.613 (0.572, 0.654) | 14.38 | ≥1.00 | 34.65 | 79.74 |
| MCIc vs NC (n=427) | 0.767 (0.724, 0.807) | 41.72 | ≥1.00 | 61.98 | 79.74 |
| MCIc vs MCIs (n=375) | 0.667 (0.617, 0.715) | 27.34 | ≥1.00 | 61.98 | 65.35 |
| **Hippocampal volume (ml)** |  |  |  |  |  |
| M-AD vs NC (n=421) | 0.916 (0.885, 0.940) | 68.88 | ≤5.38 | 82.61 | 86.27 |
| M-AD vs MCI (n=490) | 0.775 (0.735, 0.811) | 43.58 | ≤5.14 | 74.78 | 68.80 |
| M-AD vs MCIs (n=369) | 0.824 (0.782, 0.862) | 53.52 | ≤5.14 | 74.78 | 78.74 |
| M-AD vs MCIc (n=236) | 0.670 (0.607, 0.730) | 30.03 | ≤4.65 | 55.65 | 74.38 |
| MCI vs NC (n=681) | 0.699 (0.663, 0.734) | 31.65 | ≤5.81 | 61.07 | 70.59 |
| MCIs vs NC (n=560) | 0.627 (0.586, 0.668) | 20.74 | ≤5.93 | 54.72 | 66.01 |
| MCIc vs NC (n=427) | 0.850 (0.813, 0.883) | 58.29 | ≤5.61 | 81.82 | 76.47 |
| MCIc vs MCIs (n=375) | 0.729 (0.681, 0.774) | 42.45 | ≤5.61 | 81.82 | 60.63 |
| **Hippocampal fraction (%)** |  |  |  |  |  |
| M-AD vs NC (n=421) | 0.916 (0.886, 0.941) | 69.41 | ≤0.406 | 88.70 | 80.72 |
| M-AD vs MCI (n=490) | 0.763 (0.722, 0.800) | 40.29 | ≤0.364 | 66.96 | 73.33 |
| M-AD vs MCIs (n=369) | 0.814 (0.770, 0.852) | 49.40 | ≤0.367 | 68.70 | 80.71 |
| M-AD vs MCIc (n=236) | 0.655 (0.591, 0.716) | 26.20 | ≤0.354 | 61.74 | 64.46 |
| MCI vs NC (n=681) | 0.701 (0.666, 0.736) | 35.17 | ≤0.414 | 57.07 | 78.10 |
| MCIs vs NC (n=560) | 0.632 (0.591, 0.672) | 23.77 | ≤0.414 | 45.67 | 78.10 |
| MCIc vs NC (n=427) | 0.847 (0.809, 0.880) | 59.25 | ≤0.410 | 80.17 | 79.08 |
| MCIc vs MCIs (n=375) | 0.716 (0.667, 0.761) | 37.61 | ≤0.409 | 79.34 | 58.27 |
| **ILV volume (ml)** |  |  |  |  |  |
| M-AD vs NC (n=421) | 0.926 (0.897, 0.949) | 70.72 | ≥3.12 | 88.70 | 82.03 |
| M-AD vs MCI (n=490) | 0.810 (0.773, 0.844) | 48.87 | ≥3.40 | 80.87 | 68.00 |
| M-AD vs MCIs (n=369) | 0.854 (0.813, 0.888) | 56.66 | ≥3.04 | 91.30 | 65.35 |
| M-AD vs MCIc (n=236) | 0.719 (0.657, 0.775) | 37.07 | ≥3.38 | 80.87 | 56.20 |
| MCI vs NC (n=681) | 0.638 (0.601, 0.674) | 24.43 | ≥3.12 | 42.40 | 82.03 |
| MCIs vs NC (n=560) | 0.585 (0.543, 0.626) | 16.29 | ≥2.68 | 53.54 | 62.75 |
| MCIc vs NC (n=427) | 0.749 (0.705, 0.790) | 42.36 | ≥3.12 | 60.33 | 82.03 |
| MCIc vs MCIs (n=375) | 0.664 (0.614, 0.712) | 29.07 | ≥2.97 | 65.29 | 63.78 |
| **ILV fraction (%)** |  |  |  |  |  |
| M-AD vs NC (n=421) | 0.946 (0.920, 0.966) | 74.52 | ≥0.219 | 94.78 | 79.74 |
| M-AD vs MCI (n=490) | 0.830 (0.794, 0.862) | 51.22 | ≥0.232 | 86.96 | 64.27 |
| M-AD vs MCIs (n=369) | 0.875 (0.837, 0.907) | 59.09 | ≥0.229 | 87.83 | 71.26 |
| M-AD vs MCIc (n=236) | 0.735 (0.674, 0.791) | 34.89 | ≥0.232 | 86.96 | 47.93 |
| MCI vs NC (n=681) | 0.647 (0.609, 0.683) | 24.41 | ≥0.211 | 48.27 | 76.14 |
| MCIs vs NC (n=560) | 0.590 (0.548, 0.631) | 16.49 | ≥0.213 | 39.37 | 77.12 |
| MCIc vs NC (n=427) | 0.765 (0.722, 0.805) | 41.74 | ≥0.216 | 63.64 | 78.10 |
| MCIc vs MCIs (n=375) | 0.675 (0.626, 0.723) | 26.63 | ≥0.229 | 55.37 | 71.26 |

AUC: area under the curve; CI: confidence interval; ILV, inferior lateral ventricle; MTA-scale: visual rating of medial temporal lobe atrophy (average score of left and right hemispheres); vs: versus.

**eTable 11.** ROC curve analyses for differentiating different diagnoses with SMRI indexes on the males subgroup.

|  | AUC (95% CI) | Youden index (%) | Optimal threshold | Sensitivity (%) | Specificity (%) |
| --- | --- | --- | --- | --- | --- |
| **QMTA** |  |  |  |  |  |
| M-AD vs NC (n=391) | 0.975 (0.954, 0.988) | 84.66 | ≥0.711 | 90.80 | 93.86 |
| M-AD vs MCI (n=672) | 0.834 (0.803, 0.861) | 52.71 | ≥0.824 | 81.60 | 71.12 |
| M-AD vs MCIs (n=482) | 0.898 (0.867, 0.923) | 62.91 | ≥0.722 | 90.18 | 72.73 |
| M-AD vs MCIc (n=353) | 0.726 (0.676, 0.772) | 36.86 | ≥0.821 | 81.60 | 55.26 |
| MCI vs NC (n=737) | 0.746 (0.713, 0.777) | 39.85 | ≥0.553 | 66.60 | 73.25 |
| MCIs vs NC (n=547) | 0.673 (0.632, 0.712) | 29.36 | ≥0.553 | 56.11 | 73.25 |
| MCIc vs NC (n=418) | 0.869 (0.833, 0.900) | 61.23 | ≥0.629 | 75.26 | 85.96 |
| MCIc vs MCIs (n=509) | 0.727 (0.686, 0.765) | 35.19 | ≥0.674 | 68.42 | 66.77 |
| **MTA-scale (average)** |  |  |  |  |  |
| M-AD vs NC (n=391) | 0.937 (0.908, 0.959) | 72.64 | ≥1.50 | 87.12 | 85.53 |
| M-AD vs MCI (n=672) | 0.779 (0.745, 0.810) | 44.11 | ≥2.00 | 67.48 | 76.62 |
| M-AD vs MCIs (n=482) | 0.838 (0.802, 0.870) | 53.06 | ≥2.00 | 67.48 | 85.58 |
| M-AD vs MCIc (n=353) | 0.679 (0.628, 0.728) | 29.06 | ≥2.00 | 67.48 | 61.58 |
| MCI vs NC (n=737) | 0.726 (0.693, 0.758) | 35.95 | ≥1.00 | 60.51 | 75.44 |
| MCIs vs NC (n=547) | 0.665 (0.624, 0.705) | 25.60 | ≥1.00 | 50.16 | 75.44 |
| MCIc vs NC (n=418) | 0.829 (0.789, 0.864) | 53.33 | ≥1.00 | 77.89 | 75.44 |
| MCIc vs MCIs (n=509) | 0.687 (0.645, 0.727) | 27.74 | ≥1.00 | 77.89 | 49.84 |
| **Hippocampal volume (ml)** |  |  |  |  |  |
| M-AD vs NC (n=391) | 0.896 (0.862, 0.925) | 66.34 | ≤5.81 | 77.30 | 89.04 |
| M-AD vs MCI (n=672) | 0.739 (0.704, 0.772) | 37.56 | ≤5.95 | 80.98 | 56.58 |
| M-AD vs MCIs (n=482) | 0.805 (0.767, 0.839) | 48.77 | ≤5.81 | 77.30 | 71.47 |
| M-AD vs MCIc (n=353) | 0.629 (0.576, 0.680) | 22.24 | ≤5.14 | 49.08 | 73.16 |
| MCI vs NC (n=737) | 0.696 (0.661, 0.729) | 31.71 | ≤6.31 | 61.10 | 70.61 |
| MCIs vs NC (n=547) | 0.634 (0.593, 0.675) | 23.78 | ≤6.37 | 55.80 | 67.98 |
| MCIc vs NC (n=418) | 0.799 (0.758, 0.837) | 48.16 | ≤5.82 | 60.00 | 88.16 |
| MCIc vs MCIs (n=509) | 0.687 (0.645, 0.728) | 30.85 | ≤5.82 | 60.00 | 70.85 |
| **Hippocampal fraction (%)** |  |  |  |  |  |
| M-AD vs NC (n=391) | 0.918 (0.887, 0.944) | 71.07 | ≤0.369 | 81.60 | 89.47 |
| M-AD vs MCI (n=672) | 0.753 (0.719, 0.786) | 38.57 | ≤0.369 | 81.60 | 56.97 |
| M-AD vs MCIs (n=482) | 0.823 (0.786, 0.856) | 50.25 | ≤0.369 | 81.60 | 68.65 |
| M-AD vs MCIc (n=353) | 0.636 (0.584, 0.687) | 22.93 | ≤0.331 | 57.67 | 65.26 |
| MCI vs NC (n=737) | 0.698 (0.663, 0.731) | 33.32 | ≤0.367 | 41.65 | 91.67 |
| MCIs vs NC (n=547) | 0.626 (0.584, 0.667) | 21.14 | ≤0.370 | 31.66 | 89.47 |
| MCIc vs NC (n=418) | 0.818 (0.778, 0.854) | 53.77 | ≤0.367 | 62.11 | 91.67 |
| MCIc vs MCIs (n=509) | 0.705 (0.663, 0.744) | 32.74 | ≤0.366 | 61.58 | 71.16 |
| **ILV volume (ml)** |  |  |  |  |  |
| M-AD vs NC (n=391) | 0.951 (0.925, 0.970) | 76.85 | ≥4.33 | 86.50 | 90.35 |
| M-AD vs MCI (n=672) | 0.811 (0.779, 0.840) | 50.35 | ≥4.34 | 86.50 | 63.85 |
| M-AD vs MCIs (n=482) | 0.869 (0.836, 0.898) | 61.47 | ≥4.42 | 84.66 | 76.80 |
| M-AD vs MCIc (n=353) | 0.713 (0.662, 0.759) | 33.80 | ≥5.32 | 63.80 | 70.00 |
| MCI vs NC (n=737) | 0.707 (0.673, 0.740) | 32.66 | ≥3.73 | 56.78 | 75.88 |
| MCIs vs NC (n=547) | 0.644 (0.602, 0.684) | 25.48 | ≥3.39 | 61.44 | 64.04 |
| MCIc vs NC (n=418) | 0.814 (0.774, 0.850) | 50.26 | ≥3.88 | 70.00 | 80.26 |
| MCIc vs MCIs (n=509) | 0.693 (0.651, 0.732) | 32.73 | ≥4.23 | 60.00 | 72.73 |
| **ILV fraction (%)** |  |  |  |  |  |
| M-AD vs NC (n=391) | 0.947 (0.920, 0.967) | 72.83 | ≥0.290 | 78.53 | 94.30 |
| M-AD vs MCI (n=672) | 0.814 (0.782, 0.842) | 49.67 | ≥0.289 | 79.14 | 70.53 |
| M-AD vs MCIs (n=482) | 0.870 (0.837, 0.899) | 59.53 | ≥0.264 | 87.12 | 72.41 |
| M-AD vs MCIc (n=353) | 0.718 (0.668, 0.765) | 33.89 | ≥0.321 | 68.10 | 65.79 |
| MCI vs NC (n=737) | 0.711 (0.676, 0.743) | 32.94 | ≥0.203 | 75.05 | 57.89 |
| MCIs vs NC (n=547) | 0.649 (0.607, 0.689) | 26.73 | ≥0.205 | 67.08 | 59.65 |
| MCIc vs NC (n=418) | 0.814 (0.773, 0.850) | 51.14 | ≥0.241 | 72.63 | 78.51 |
| MCIc vs MCIs (n=509) | 0.692 (0.650, 0.732) | 33.64 | ≥0.246 | 70.00 | 63.64 |

AUC: area under the curve; CI: confidence interval; ILV, inferior lateral ventricle; MTA-scale: visual rating of medial temporal lobe atrophy (average score of left and right hemispheres); vs: versus.

**eTable 12.** Demographic characteristics, brain structure, and performance across NC A-, MCIs A- and MCIc A+ subgroups

|  | NC A- (n=325) | MCIs A- (n=274) | MCIc A+ (n=165) | *P* (NC A- vs MCIc A+) | *P* (MCIs A- vs MCIc A+) |
| --- | --- | --- | --- | --- | --- |
| Age (years), median(IQR) | 72.00 (68.00, 77.00) | 71.00 (65.00, 77.00) | 74.00 (69.30, 78.30) | 0.048 | 0.005 |
| Male, n (%) | 136 (42.7) | 147 (53.6) | 98 (59.39) | 0.320 | 0.015 |
| Education (years), median(IQR) | 16.00 (15.00, 18.00) | 16.00 (14.00, 18.00) | 16.00 (14.00, 18.00) | 0.117 | 0.478 |
| MMSE, median(IQR) | 29.00 (29.00, 30.00) | 28.00 (28.00, 29.00) | 27.00 (26.00, 29.00) | <0.001 | <0.001 |
| FAQ, median(IQR) | 0.00 (0.00, 0.00) | 1.00 (0.00, 2.00) | 4.00 (1.00,8.00) | <0.001 | <0.001 |
| CDR, median(IQR) | 0.00 (0.00, 0.00) | 0.50 (0.50, 0.50) | 0.50 (0.50, 0.50) | <0.001 | 0.799 |
| CDR-SOB, median(IQR) | 0.00 (0.00, 0.00) | 1.00 (0.50, 1.50) | 1.50 (1.00, 2.50) | <0.001 | <0.001 |
| ADAS-cog, median(IQR) | 7.00 (4.00, 9.33) | 8.00 (6.00, 11.42) | 17.33 (12.00, 22.00) | <0.001 | <0.001 |
| CSF Aβ_42_ (pg/ml), median(IQR) | 1529.00 (1182.00, 2022.00) | 1427.50 (1106.5, 1892.50) | 611.00 (516.00, 716.30) | 0.040 | <0.001 |
| CSF P-tau^181^ (pg/ml), median(IQR) | 19.00 (15.43, 24.79) | 19.67 (15.25, 26.00) | 32.97 (26.26, 48.18) | 0.484 | <0.001 |
| CSF T-tau (pg/ml),median(IQR) | 215.45 (176.90, 270.95) | 228.90 (176.00, 287.00) | 334.95 (266.43, 452.65) | 0.416 | <0.001 |
| HV (ml), mean ± SD | 6.36 ± 0.74 | 6.17 ± 0.90 | 5.43 ± 0.85 | <0.001 | <0.001 |
| HF, mean ± SD | 0.433 ± 0.046 | 0.413 ± 0.061 | 0.361 ± 0.054 | <0.001 | <0.001 |
| ILV volume(ml), median(IQR) | 2.73 (2.33, 3.23) | 3.05 (2.47, 3.83) | 3.97 (3.02, 5.05) | <0.001 | <0.001 |
| ILV fraction, median(IQR) | 0.186 (0.163, 0.211) | 0.203 (0.169, 0.245) | 0.264 (0.204, 0.320) | <0.001 | <0.001 |
| QMTA, median(IQR) | 0.432 (0.373, 0.503) | 0.473 (0.388, 0.622) | 0.718 (0.563, 0.927) | <0.001 | <0.001 |
| MTA-avg,median(IQR) | 1.00 (0.50, 1.00) | 1.00 (1.00, 2.00) | 1.50 (1.00, 2.50) | <0.001 | <0.001 |

IQR: interquartile rang; FAQ: Functional Activities Questionnaire; CDR: Clinical Dementia Rating; CDR-SOB: Clinical Dementia Rating sum of boxes; ADAS-cog: Alzheimer’s disease assessment scale-cognitive; HV: the absolute volumes of hippocampus; HF: Hippocampal fraction; ILV: Inferior lateral ventricle; avg: average.

**eTable 13-1.** Cut-off values of QMTA and MTA in distinguishing MCIc from MCIs

|  | Age Group | Cutoffs | Sen (%) | Spec (%) | FPR (%) | FNR (%) | PPV (%) | NPV (%) |
| --- | --- | --- | --- | --- | --- | --- | --- | --- |
| QMTA | Total | 0.635 | 67.20 | 66.49 | 33.51 | 32.80 | 78.88 | 52.12 |
|  | ≤75yrs | 0.635 | 58.10 | 75.96 | 24.04 | 41.90 | 54.17 | 78.75 |
|  | >75yrs | 0.635 | 79.55 | 49.76 | 50.24 | 20.45 | 50.24 | 79.23 |
| MTA | Total | 1.0 | 95.50 | 12.91 | 87.09 | 4.50 | 37.31 | 84.09 |
|  | ≤75yrs | 1.0 | 94.41 | 16.12 | 83.88 | 5.59 | 35.50 | 85.51 |
|  | >75yrs | 1.0 | 96.97 | 7.25 | 92.75 | 3.03 | 40.00 | 78.95 |

AUC: Area under curve, CI: Confidence intervals, Sen: sensitivity, Spec: specificity, FPR: false positive rate; FNR: false negative rate, PPV: positive predictive values, NPV: negative predictive value, yrs: years.

**eTable 13-2.** Cut-off values of QMTA and MTA in distinguishing MCIc from MCIs (follow-up time > 5 years)

|  | Age Group | Cutoffs | Sen (%) | Spec (%) | FPR (%) | FNR (%) | PPV (%) | NPV (%) |
| --- | --- | --- | --- | --- | --- | --- | --- | --- |
| QMTA | Total | 0.635 | 58.79 | 69.44 | 30.56 | 41.21 | 68.79 | 59.52 |
|  | ≤75yrs | 0.635 | 46.88 | 77.78 | 22.22 | 53.13 | 67.16 | 60.16 |
|  | >75yrs | 0.635 | 75.36 | 51.11 | 48.89 | 23.63 | 70.27 | 57.50 |
| MTA | Total | 1.0 | 92.12 | 15.28 | 92.12 | 15.28 | 44.53 | 37.14 |
|  | ≤75yrs | 1.0 | 90.63 | 19.19 | 80.81 | 9.38 | 52.10 | 67.86 |
|  | >75yrs | 1.0 | 94.20 | 6.67 | 93.33 | 5.80 | 60.75 | 42.86 |

AUC: Area under curve, CI: Confidence intervals, Sen: sensitivity, Spec: specificity, FPR: false positive rate; FNR: false negative rate, PPV: positive predictive values, NPV: negative predictive value, yrs: years.
